# Artificial Intelligence and Radiologist Burnout

**DOI:** 10.1001/jamanetworkopen.2024.48714

**Published:** 2024-11-22

**Authors:** Hui Liu, Ning Ding, Xinying Li, Yunli Chen, Hao Sun, Yuanyuan Huang, Chen Liu, Pengpeng Ye, Zhengyu Jin, Heling Bao, Huadan Xue

**Affiliations:** 1 Institute of Medical Information, Chinese Academy of Medical Sciences & Peking Union Medical College, Beijing, China; 2 Radiology Department, State Key Laboratory of Complex Severe and Rare Diseases, Peking Union Medical College Hospital, Chinese Academy of Medical Science & Peking Union Medical College, Beijing, China; 3 National Center for Quality Control of Radiology, Beijing, China; 4 Key Laboratory of Mental Health, Institute of Psychology, Chinese Academy of Sciences, Beijing, China; 5 Psychological Health Center, Beijing United Family Hospital, Beijing, China; 6 National Centre for Non-Communicable Disease Control and Prevention, Chinese Centre for Disease Control and Prevention, Beijing, China

## Abstract

**IMPORTANCE:**

Understanding the association of artificial intelligence (AI) with physician burnout is crucial for fostering a collaborative interactive environment between physicians and AI.

**OBJECTIVE:**

To estimate the association between AI use in radiology and radiologist burnout.

**DESIGN, SETTING, AND PARTICIPANTS:**

This cross-sectional study conducted a questionnaire survey between May and October 2023, using the national quality control system of radiology in China. Participants included radiologists from 1143 hospitals. Radiologists reporting regular or consistent AI use were categorized as the AI group. Statistical analysis was performed from October 2023 to May 2024.

**EXPOSURE:**

AI use in radiology practice.

**MAIN OUTCOMES AND MEASURES:**

Burnout was defined by emotional exhaustion (EE) or depersonalization according to the Maslach Burnout Inventory. Workload was assessed based on working hours, number of image interpretations, hospital level, device type, and role in the workflow. AI acceptance was determined via latent class analysis considering AI-related knowledge, attitude, confidence, and intention. Propensity score–based mixed-effect generalized linear logistic regression was used to estimate the associations between AI use and burnout and its components. Interactions of AI use, workload, and AI acceptance were assessed on additive and multiplicative scales.

**RESULTS:**

Among 6726 radiologists included in this study, 2376 (35.3%) were female and 4350 (64.7%) were male; the median (IQR) age was 41 (34-48) years; 3017 were in the AI group (1134 [37.6%] female; median [IQR] age, 40 [33-47] years) and 3709 in the non-AI group (1242 [33.5%] female; median [IQR] age, 42 [34-49] years). The weighted prevalence of burnout was significantly higher in the AI group compared with the non-AI group (40.9% vs 38.6%; *P* < .001). After adjusting for covariates, AI use was significantly associated with increased odds of burnout (odds ratio [OR], 1.20; 95% CI, 1.10-1.30), primarily driven by its association with EE (OR, 1.21; 95% CI, 1.10-1.34). A dose-response association was observed between the frequency of AI use and burnout (*P *for trend < .001). The associations were more pronounced among radiologists with high workload and lower AI acceptance. A significant negative interaction was noted between high AI acceptance and AI use.

**CONCLUSIONS AND RELEVANCE:**

In this cross-sectional study of radiologist burnout, frequent AI use was associated with an increased risk of radiologist burnout, particularly among those with high workload or lower AI acceptance. Further longitudinal studies are needed to provide more evidence.

## Key Points

**Question:**

Is the use of artificial intelligence (AI) in radiology practice associated with radiologist burnout?

**Findings:**

In this cross-sectional study, the use of AI was associated with burnout among radiologists, exhibiting a dose-response association. This association was particularly pronounced in radiologists with high workload and those with low AI acceptance.

**Meaning:**

These findings suggest the need for harmonious integration of AI tools with radiologists to effectively mitigate burnout in radiology practice.

## Teaser

This cross-sectional study investigates whether there is an association between artificial intelligence (AI) use in radiology and radiologist burnout in China.

## Introduction

Burnout encompasses emotional exhaustion (EE), depersonalization (DP), and a diminished sense of personal accomplishment.^1^ Physician burnout has emerged as a global issue,^2^ primarily driven by work overload, conflicts between home and work life, and job dissatisfaction.^3,4^ A previous systematic review has linked physician burnout to career disengagement, high physician turnover, and reduced quality of patient care.^5^ Radiologists exhibit higher burnout rates compared with other medical specialists. In Germany, over 75% of radiologists have reported experiencing burnout,^6^ while approximately 40% of their US counterparts report similar conditions.^7,8^ A recent analysis estimated that 83% of radiologists exhibit at least 1 symptom of burnout.^9^ Moreover, elevated burnout levels have been documented across various radiology subspecialties, including interventional, musculoskeletal, pediatric, and breast.^10,11,12,13^

The integration of artificial intelligence (AI) in radiology practice is becoming increasingly prevalent, promising to alleviate the workload of radiologists.^14^ However, recent studies have shown mixed outcomes, with nearly half of the respondents indicating that AI either increases or does not significantly impact their workload.^15,16^ Furthermore, the relationship between AI adoption and radiologist burnout remains unclear, warranting further investigation. This cross-sectional study, based on a nationwide survey in China, investigated the association between AI use and burnout by examining the complex interactions among AI use, professional characteristics, and burnout risk. The aim of this study was to propose several short-term, localized strategies that radiology practices can adopt to enhance workforce capacity and efficiency, thereby alleviating current workload challenges.

## Methods

### Study Design and Participants

From May 1 to October 31, 2023, we conducted a cross-sectional study to obtain a nationally representative sample of radiologists from secondary and higher hospitals through the National Center for Quality Control of Radiology network. The network operates as a tiered system with sentinel surveillance across all 31 provincial units and Xinjiang Production and Construction Corps in mainland China (eAppendix 1 in Supplement 1). We selected 1143 hospitals and enrolled 1 to 5 radiologists aged 20 to 74 years from each, provided they had worked for at least 1 year before the survey.^17^ Radiologists who were pregnant or who joined the hospital after the retirement age of 60 years were excluded. Eligible radiologists were invited to anonymously complete a self-administered questionnaire via the electronic platform WJX.cn. A cover letter outlined the survey’s purpose, and the completion of the questionnaire implied consent to participate. The study was approved by the Ethics Committee of the Peking Union Medical College Hospital and adhered to the Strengthening the Reporting of Observational Studies in Epidemiology (STROBE) reporting guideline.

### Measures

The primary outcome was the prevalence of burnout, measured using the Chinese version of the 22-item Maslach Burnout Inventory-Human Services Survey (MBI-HSS). This instrument evaluates burnout across 3 dimensions: EE, DP, and personal accomplishment.^1,18^ According to the most common scoring criteria,^2^ burnout was defined as exhibiting at least 1 symptom of EE (≥27) or DP (≥10). In our data, the scale demonstrated a Cronbach α of 0.86 overall, with 0.92 for EE and 0.86 for DP.

We designed the survey questionnaire based on validated scales for AI literacy and adoption.^19^ The frequency of AI use in radiology was gauged by asking, “How frequently have you employed AI for image interpretation within the past year?” Response options included “Never,” “Infrequently,” “Regularly,” and “Consistently.”^20^ Radiologists reporting regular or consistent AI use were classified into the AI group, whereas others were classified into the non-AI group.

AI acceptance was evaluated through the Technology Acceptance Model,^21^ encompassing 4 dimensions: knowledge, confidence, attitude, and intention. Each dimension was categorized into low and high levels based on self-reported classifications and sample sizes. An overall measure of AI acceptance was derived using latent class analysis (eAppendix 2 in Supplement 1). The analysis identified 2 distinct latent classes: low acceptance and high acceptance.

Based on previous studies of risk factors for burnout, we assessed workload, psychological factors of job satisfaction, and personal and professional characteristics. Workload was evaluated through working hours on image interpretation, the volume of image interpretation, device type, role in the reporting workflow, and hospital level. Each factor was assigned 0 points for low workload and 1 point for high workload, resulting in an overall workload score ranging from 0 to 5 (eAppendix 2 in Supplement 1). Radiologists were categorized into 3 classes: low (0-2 points), medium (3 points), and high (4-5 points).

Psychological factors were measured using the Gallup Q12 Employee Engagement scale,^22^ which assesses perceived control, spiritual rewards, work values, organizational support, and coworker support (eAppendix 3 in Supplement 1), demonstrating a Cronbach α of 0.94 in our data. Additionally, personal and professional characteristics were gathered via a self-designed questionnaire, encompassing age, sex, education level, geographic location, marital status, parental status, and average monthly salary, along with years of full-time experience in radiology and professional title and/or position level.

### Statistical Analysis

Continuous variables were summarized as median with IQR, while categorical variables were presented as frequencies. Differences between groups were assessed using the Mann-Whitney *U* test or *χ^2^* test. Generalized linear regression model was used to examine the association between AI use and burnout, adjusting for personal and professional characteristics, workload score, AI acceptance, and psychological factors. We used random effects by provincial units to account for clustering of the multilayer data. AI use was analyzed as both a categorical and continuous variable to illustrate the dose-response association.

To address nonrandomized group assignments, propensity-score methods were used to mitigate confounding effects. Individual propensities for AI use were estimated using a multivariable logistic regression model, incorporating the same covariates as the explanatory model. Three propensity score–based strategies were implemented (eAppendix 4 in Supplement 1): (1) a model with inverse probability weighting (IPW), estimated using predicted probabilities from the propensity-score model; (2) a model based on matched samples created through the nearest neighbor propensity-score matching; and (3) a model that included the propensity score as an additional covariate. The primary analysis focused on models using the IPW strategy.

Stratified analyses were conducted by demographic status, hospital type, workload score, and AI acceptance. To assess the interaction among workload score, AI acceptance, and AI use on burnout, joint association analyses classified radiologists into subgroups based on workload score and AI acceptance. Given that the odds ratios (ORs) may be distorted when diseases are not rare, adjusted prevalence ratios (PRs) were calculated for joint exposure to AI use in conjunction with high workload or low AI acceptance using log-binomial regression.^23^ Interaction measures were determined on both additive and multiplicative scales, with additive interaction estimated by the relative excess risk due to interaction (RERI) and multiplicative interaction by the ratio of the PR (RPR). Furthermore, we plotted the burnout prediction probabilities of burnout derived from models incorporating interaction terms to illustrate these interactions. Sensitivity analyses included repeating propensity score–based models by substituting overall workload score with individual workload factors and evaluating regional variations in associations by categorizing radiologists into 4 geographic groups.

Statistical analysis was performed from October 2023 to May 2024. All models were fitted using the glmmTMB package in R statistical software version 4.2.2 (R Foundation for Statistical Computing). The Hochberg method was applied to adjust *P* values for multiple testing.^24^ All tests were 2-sided with a significance threshold of *P* < .05.

## Results

Table 1 presents the personal and professional characteristics of the eligible radiologists. Among the 7520 radiologists invited, 6726 responded, yielding a response rate of 89.4% (eFigure 1 in Supplement 1). The median (IQR) age of the participants was 41 (34-48) years; 2376 (35.3%) were female and 4350 (64.7%) were male. Most (4749 [70.6%]) were from eastern and western China, 5716 (85.0%) were married, and 5493 (81.7%) had children. The median (IQR) duration of practice in radiology was 16 (8-25) years, and 3214 (47.8%) provided both first and second opinions in the reporting workflow. There were statistically significant differences in age, having children, marital status, and income level between respondents and nonrespondents (eTable 1 and 2 in Supplement 1).

**Table 1.** Personal and Professional Characteristics of Radiologists

Characteristics	Radiologists, No. (%)			Radiologists after inverse probability weighting, %	
	Total (N = 6726)	Crude			
		AI group (n = 3017)	Non-AI group (n = 3709)	AI group	Non-AI group
Personal					
Age, median (IQR), y	41 (34-48)	40 (33-47)	42 (34-49)	41 (34-49)	41 (34-48)
Sex					
Male	4350 (64.7)	1883 (62.4)	2467 (66.5)	65.2	65.1
Female	2376 (35.3)	1134 (37.6)	1242 (33.5)	34.8	34.9
Have children	5493 (81.7)	2401 (79.6)	3092 (83.4)	82.6	82.4
Geographic region					
Eastern	2686 (39.9)	1327 (44.0)	1359 (36.6)	41.0	41.0
Central	1147 (17.1)	456 (15.1)	691 (18.6)	16.4	16.7
Western	2063 (30.7)	919 (30.5)	1144 (30.8)	30.6	30.2
Northeast	830 (12.3)	315 (10.4)	515 (13.9)	12.0	12.3
Relationship status					
Single	905 (13.5)	436 (14.5)	469 (12.6)	12.4	13.2
Married	5716 (85.0)	2536 (84.1)	3180 (85.7)	86.1	85.3
Others^a^	105 (1.5)	45 (1.5)	60 (1.6)	1.4	1.5
Monthly income, <5000	5447 (80.9)	2132 (70.7)	3315 (89.4)	80.5	80.4
Education levels, low^b^	5502 (81.8)	2127 (70.5)	3375 (91.0)	81.7	82.0
Professional					
Specialty, breast, chest, or blood vessel	5513 (82.0)	2458 (81.5)	3055 (82.4)	84.2	80.9
Years in practice, median (IQR), y	16 (8-25)	15 (8-24)	17 (9-26)	17 (9-26)	16 (9-26)
Workload score, median (IQR)	3 (2-3)	3 (2-4)	2 (2-3)	3 (2-3)	3 (2-3)
Hours worked per week, median (IQR), h	42 (40-50)	45 (40-50)	40 (40-50)	44 (40-50)	42 (40-50)
No. of images per day, median (IQR)	115 (72-180)	131 (90-208)	100 (60-157)	120 (75-183)	112 (72-182)
Tertiary hospitals	3988 (59.3)	2376 (78.8)	1612 (43.5)	60.1	59.6
Senior professional title	2500 (37.2)	1229 (40.7)	1271 (34.3)	41.9	37.5
Main working in practice					
Radiograph	2526 (37.6)	913 (30.3)	1613 (43.9)	36.1	36.3
Computerized tomography	3754 (55.8)	1955 (64.8)	1799 (48.5)	57.1	56.5
Magnetic resonance imaging	446 (6.6)	149 (4.9)	297 (8.0)	6.8	7.2
Role in the workflow					
Initial opinion	1774 (26.4)	759 (25.2)	1015 (27.4)	25.2	26.0
Second opinion	1738 (25.8)	982 (32.6)	756 (20.4)	29.9	24.8
Both	3214 (47.8)	1276 (42.3)	1938 (52.3)	44.9	49.3
AI acceptance, high	5222 (77.6)	2645 (71.3)	2577 (85.4)	78.0	77.7
Attitude toward AI, positive	6253 (93.0)	2930 (97.1)	3323 (89.6)	97.1	90.3
Knowledge of AI, familiar	3047 (45.3)	1582 (52.4)	1465 (39.5)	48.7	43.6
Confidence on AI, positive	4482 (66.6)	2223 (73.7)	2259 (60.9)	72.4	61.9
Motivation to learn AI, positive	5254 (78.1)	2702 (72.9)	2552 (84.6)	78.7	78.3

Abbreviation: AI, artificial intelligence.

^a^ Others include divorce, separation, widowed, or widower.

^b^ Bachelor’s degree and below.

There were 3017 radiologists regularly or consistently using AI in their practice. These radiologists were younger (median [IQR] ages: 40 [33-47] vs 42 [34-49] years; *P* < .001), more likely to be female (1134 [37.6%] vs 1242 [33.5%]; *P* < .001), more likely from eastern China (1327 [44.0%] vs 1359 [36.6%]; *P* < .001), and had higher educational levels (3890 [29.5%] vs 334 [9.0%]; *P* < .001) compared with those in the non-AI group. Additionally, the proportion of radiologists primarily practicing computed tomography reading was higher in the AI group compared with the non-AI group (1955 [64.8%] vs 1799 [48.5%]; *P* < .001). Radiologists in the AI group had fewer median (IQR) full-time working years than their counterparts (15 [8-24] vs 17 [9-26] years; *P* < .001), but they worked more median (IQR) hours per week (45 [40-50] vs 40 [40-50] hours; *P* = .001) and interpreted more images per day (131 [90-208] vs 100 [60-157]; *P* < .001). After adjustment based on propensity score, differences in the characteristics between the AI and non-AI groups were significantly reduced or eliminated (Table 1; eTable 3 in Supplement 1).

Table 2 compares burnout, its components, and psychological factors of job satisfaction between the AI and the non-AI groups. Overall, 2675 radiologists were classified as experiencing burnout. The crude prevalence of burnout among participants was 39.8%, with EE reported by 34.4% and DP by 18.1%. The weighted prevalence of burnout was higher in the AI group than in the non-AI group (40.9% vs 38.6%; *P* = .006), primarily due to differences in EE (35.4% vs 33.2%; *P* = .006). The weighted prevalence of DP was similar between groups (18.4% vs 18.1%; *P* = .64). Additionally, the AI group exhibited higher weighted prevalence of burnout across various subgroups (eTable 4 in Supplement 1).

**Table 2.** Burnout and Career Satisfaction by AI Use

Variable	Total (N = 6726)	Crude, No. (%)		Inverse probability weighting, %	
		AI group (n = 3017)	Non-AI group (n = 3709)	AI group (n = 3017)	Non-AI group (n = 3709)
Burnout^a^	2675 (39.8)	1293 (42.9)	1382 (37.3)	40.9	38.6
Burnout measurement					
Emotional exhaustion					
Mean (SD)	21.3 (14.0)	22.5 (13.8)	20.4 (14.1)	22.0 (20.6)	20.9 (18.9)
Median (IQR)	19 (10-31)	21 (11,33)	17 (9-30)	19 (10-32)	18 (9-30)
Proportion of ≥27	2311 (34.4)	1140 (37.8)	1171 (31.6)	35.4	33.2
Depersonalization					
Mean (SD)	4.9 (6.1)	4.9 (5.9)	4.9 (6.2)	4.9 (8.9)	4.9 (8.2)
Median (IQR)	3 (1-7)	3 (1-7)	3 (0-7)	3 (1-7)	3 (1-7)
Proportion of ≥10	1214 (18.1)	545 (18.1)	669 (18.0)	18.4	18.1
Autonomy, control, and meaning in work^b^					
Autonomy, agree	5242 (77.9)	2376 (78.8)	2866 (77.3)	77.9	78.0
Control, agree	3740 (55.6)	1800 (59.7)	1940 (52.3)	56.1	55.7
Meaningful, agree	4337 (64.5)	2009 (66.6)	2328 (62.8)	64.8	64.7
Supporting circumstance^c^					
Supporting from staff, agree	3893 (57.9)	2168 (71.9)	2563 (69.1)	70.1	70.2
Supporting from organization, agree	4031 (59.9)	1915 (63.5)	2116 (57.1)	60.0	59.8

Abbreviation: AI, artificial intelligence.

^a^ Burnout is defined as emotional exhaustion score of at least 27, or depersonalization score of at least 10.

^b^ Questions are from the Q3, Q4, Q5, Q8, and Q9 of the Gallup Q12 Employee Engagement scale.

^c^ Questions are from the Q5 and Q9 of the Gallup Q12 Employee Engagement scale.

A majority of radiologists reported having autonomy in their practice (77.9%), with no significant difference between the 2 groups. A higher proportion of radiologists in the AI group felt their opinions were valued in practice (59.7%) and that their work was meaningful (66.6%), compared with those in the non-AI group. Additionally, satisfaction with organizational support (1915 [63.5%] vs 2116 [57.1%]; *P* = .01) and staff (2168 [71.9%] vs 2563 [69.1%]; *P* < .001) was higher in the AI group.

In crude regression analysis (Table 3), radiologists using AI were more likely to experience burnout than those who did not (OR, 1.26; 95% CI, 1.14-1.40). This association remained significant after adjustment for covariates (adjusted OR, 1.18; 95% CI, 1.05-1.33). Dose-response analysis indicated that the odds of burnout increased with the frequency of AI use (regularly: OR, 1.17; 95% CI, 1.04-1.32; consistently: OR, 1.39; 95% CI, 1.09-1.75). In the primary analysis with IPW, AI use was significantly associated with higher odds of burnout (OR, 1.20; 95% CI, 1.10-1.30). AI use was significantly associated with EE (OR, 1.21; 95% CI, 1.10-1.34), but not with DP.

**Table 3.** Association of AI Use With Burnout in Crude, Multivariable, and Propensity Score Analysis

	Burnout		Emotional exhaustion		Depersonalization	
	OR (95% CI)	*P* value	OR (95% CI)	*P* value	OR (95% CI)	*P* value
Crude analysis (yes vs no)	1.26 (1.14-1.40)	<.001	1.31 (1.18-1.45)	<.001	1.01 (0.89-1.15)	.86
Multivariable analysis (yes vs no)^a^	1.18 (1.05-1.33)	.004	1.21 (1.08-1.37)	.002	1.03 (0.90-1.19)	.66
Multivariable analysis (vs no)^b^						
Regularly	1.17 (1.04-1.32)	.008	1.19 (1.05-1.34)	.005	0.99 (0.86-1.14)	.87
Consistently	1.39 (1.09-1.75)	.008	1.41 (1.11-1.80)	.005	1.43 (1.09-1.88)	.02
Multivariable analysis (continuous)^c^	1.18 (1.07-1.30)	<.001	1.19 (1.08-1.31)	<.001	1.09 (0.97-1.22)	.14
Propensity-score analyses						
With IPW (yes vs no)^d^	1.20 (1.10-1.30)	<.001	1.21 (1.10-1.34)	<.001	1.11 (0.99-1.23)	.06
With matching (yes vs no)^e^	1.28 (1.12-1.46)	<.001	1.31 (1.14-1.50)	<.001	1.08 (0.92-1.26)	.36
Adjusted for propensity score (yes vs no)^f^	1.18 (1.05-1.33)	.005	1.21 (1.07-1.36)	.002	1.00 (0.87-1.15)	.99

Abbreviations: AI, artificial intelligence; IPW, inverse probability weighting; OR, odds ratio.

^a^ Result is the adjusted OR from the multivariable logistic regression adjusting for covariates and random effect of provinces. The analysis included 6726 radiologists.

^b^ Frequency of using AI in practice was regarded as categorical variable in the multivariable logistic regression adjusting for covariates and random effect of provinces. The analysis included 6726 radiologists. *P* value was corrected by Hochberg method.

^c^ Frequency of using AI in practice was regarded as continuous variable in the multivariable logistic regression adjusting for covariates and random effect of provinces. The analysis included 6726 radiologists.

^d^ Result is the adjusted OR from the multivariable logistic regression with IPW, adjusting for covariates and random effect of provinces. The analysis included 6726 radiologists.

^e^ Frequency of using AI in practice was regarded as categorical variable in the multivariable logistic regression adjusting for covariates. The analysis included 3978 matching radiologists.

^f^ Result is the adjusted OR from the multivariable logistic regression adjusting for covariates, the propensity score, and random effect of provinces. The analysis included 6726 radiologists.

Stratified analysis (Table 4; eFigure 2 in Supplement 1) revealed significant associations between AI use and burnout or EE in most subgroups, except those with higher education levels, secondary hospital, low workload score, and high AI acceptance. No significant associations were found for DP in most subgroups. Associations were stronger among those who consistently used AI, and the dose-response association was significant in most subgroups (eTable 5, 6, and 7 in Supplement 1). Notably, radiologists with low AI acceptance had significant associations for all 3 burnout indices.

**Table 4.** Association of AI Use by Frequency With Burnout by Demographic Status and Workload and AI Acceptance

Characteristics	Burnout^a^			Emotional exhaustion			Depersonalization		
	Regularly AI use, OR (95% CI)^b^	Consistently AI use, OR (95% CI)^b^	*P* value for trend^c^	Regularly AI use, OR (95% CI)^b^	Consistently AI use, OR (95% CI)^b^	*P* value for trend^c^	Regularly AI use, OR (95% CI)^b^	Consistently AI use, OR (95% CI)^b^	*P* value for trend^c^
Sex									
Male	1.10 (0.97-1.24)	1.68 (1.24-2.28)^d^	.002	1.08 (0.95-1.23)	1.63 (1.21-2.18)^d^	.005	1.06 (0.93-1.20)	1.84 (1.40-2.41)^d^	.001
Female	1.37 (1.15-1.63)^d^	1.35 (0.96-1.91)	.001	1.38 (1.15-1.64)^d^	1.37 (0.97-1.93)	<.001	0.96 (0.79-1.18)	1.24 (0.83-1.86)	.66
Age group									
<40 y	1.23 (1.05-1.43)^d^	1.53 (1.10-2.12)^d^	<.001	1.24 (1.06-1.45)^d^	1.54 (1.11-2.14)^d^	<.001	1.03 (0.87-1.21)	1.48 (1.07-2.05)^d^	.08
≥40 y	1.16 (1.01-1.32)^d^	1.48 (1.09-2.01)^d^	.003	1.15 (1.00-1.31)^d^	1.43 (1.05-1.94)^d^	.006	1.08 (0.94-1.25)	1.72 (1.27-2.34)^d^	.005
Education level^e^									
Low	1.19 (1.07-1.33)^d^	1.57 (1.22-2.02)^d^	<.001	1.18 (1.06-1.32)^d^	1.55 (1.21-1.98)^d^	<.001	1.09 (0.97-1.23)	1.68 (1.32-2.15)^d^	<.001
High	1.26 (0.97-1.64)	1.10 (0.68-1.77)	0.24	1.33 (1.02-1.73)	1.23 (0.75-2.10)	.08	0.90 (0.69-1.20)	1.19 (0.73-1.95)	.91
Hospital type									
Secondary	1.14 (0.96-1.34)	1.42 (0.89-2.28)	.04	1.06 (0.89-1.25)	1.36 (0.85-2.20)	.24	1.06 (0.89-1.27)	1.37 (0.85-2.20)	.22
Tertiary	1.23 (1.08-1.40)^d^	1.48 (1.15-1.92)^d^	<.001	1.29 (1.13-1.47)^d^	1.52 (1.18-1.96)^d^	<.001	1.06 (0.92-1.22)	1.60 (1.24-2.05)^d^	.003
Workload score									
Low score	1.09 (0.94-1.27)	1.73 (1.20-2.49)^d^	.01	1.07 (0.91-1.25)	1.53 (1.06-2.21)^d^	.06	1.06 (0.90-1.25)	1.89 (1.29-2.77)^d^	.02
Medium score	1.32 (1.11-1.58)^d^	1.24 (0.87-1.74)	.006	1.30 (1.10-1.54)^d^	1.23 (0.87-1.74)	.007	1.14 (0.95-1.37)	1.35 (0.93-1.95)	.06
High score	1.27 (1.01-1.61)	1.60 (1.00-2.56)^d^	.01	1.35 (1.07-1.69)^d^	1.74 (1.10-2.75)^d^	.002	0.94 (0.76-1.17)	1.71 (1.14-2.58)^d^	.16
AI acceptance									
Low	1.55 (1.24-1.92)^d^	2.50 (1.33-4.71)^d^	<.001	1.50 (1.21-1.86)^d^	2.68 (1.43-5.02)^d^	<.001	1.43 (1.13-1.79)^d^	1.45 (0.81-2.58)	.004
High	1.10 (0.98-1.24)	1.30 (1.02-1.65)	.01	1.12 (0.99-1.25)	1.26 (0.99-1.60)	.02	0.97 (0.86-1.10)	1.54 (1.22-1.96)^d^	.04

Abbreviations: AI, artificial intelligence; OR, odds ratio.

^a^ Burnout was defined as having at least 1 symptom of the emotional exhaustion (≥27) or depersonalization (≥10).

^b^ The regressions with inverse probability weighting were fitted by taking the frequency of AI use as categorical variable, adjusting for covariates and random effect of provinces, with the exception of stratified variables, which were mutually adjusted.

^c^ *P* for trend was calculated by the same model using frequency of AI use as continuous variable.

^d^ *P* < .05 with multiple testing correction by Hochberg method.

^e^ Education level was categorized as bachelor’s degree and below and graduate degree and above.

Joint association analysis (Figure) exhibited the highest PR of 1.66 (95% CI, 1.50 to 1.82) among radiologists with high workload scores compared with those with lower workloads. Although there was positive interaction on both additive and multiplicative scales, it was not statistically significant. Conversely, the highest PR of 1.27 (95% CI, 1.14 to 1.42) for burnout was found among radiologists with low AI acceptance, whereas the association attenuated to 1.16 (95% CI, 1.06 to 1.27) among those with high acceptance. There was a significantly negative interaction on both additive (RERI, −0.20; 95% CI, −0.35 to −0.05) and multiplicative scales (RPR, 0.84; 95% CI, 0.73 to 0.95). Similar patterns were observed for EE and DP (eFigure 3, eTable 8, and eTable 9 in Supplement 1).

**Figure.**
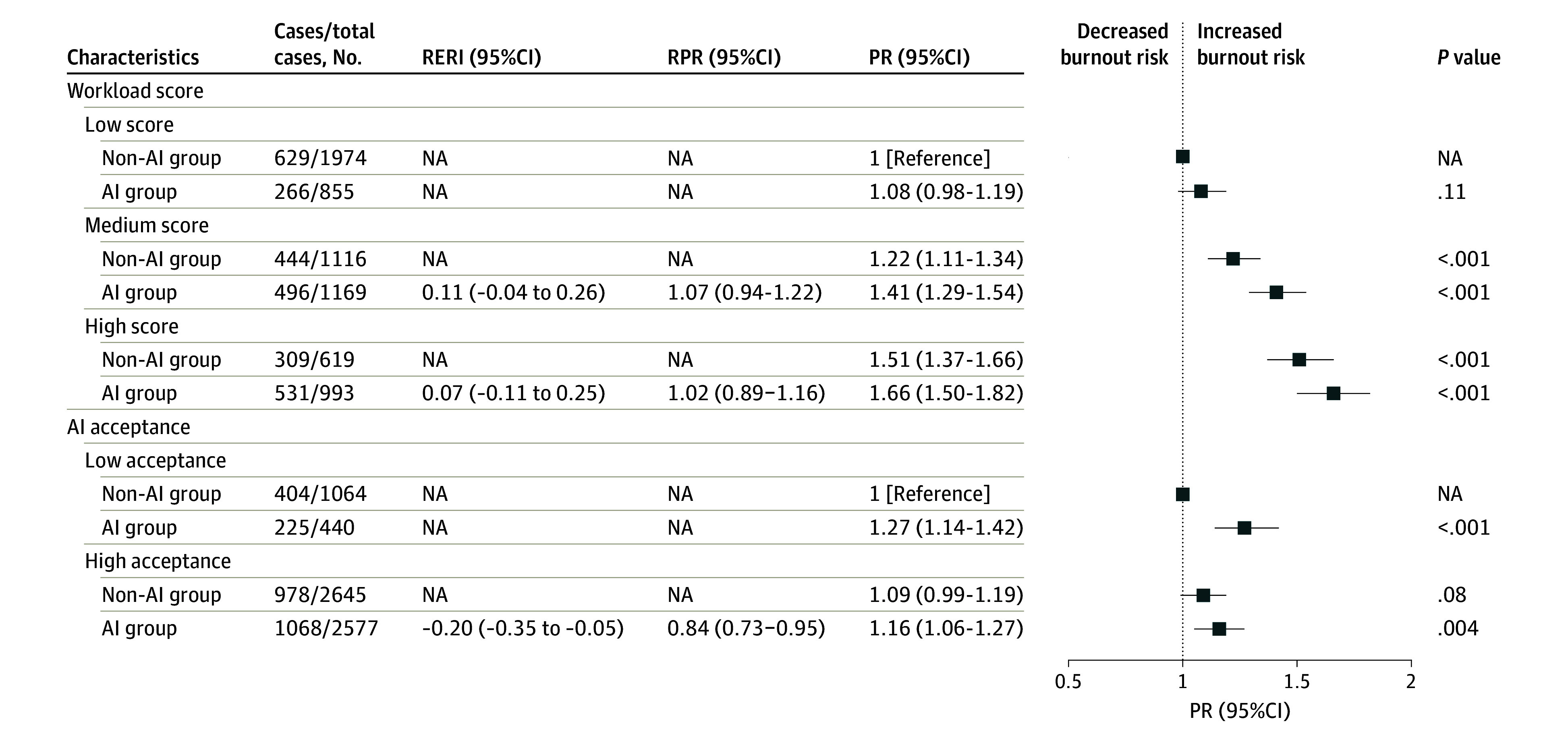
Joint Association of AI Use, Workload, and AI Acceptance With Burnout Prevalence ratios (PRs) were calculated for the joint exposure of AI use and workload score groups by binomial-log regression, adjusting for individual and professional characteristics and psychological factors. All analyses were calculated using inverse propensity weighting. Relative excess risk due to interaction (RERI) was estimated using the following formula: PR_11_ − PR_10_ − PR_01_ + 1. Ratio of prevalence risk (RPR) was estimated using PR_11_ / (PR_01_ × PR_10_). *P* value was corrected by Hochberg method. AI indicates artificial intelligence; NA, not applicable.

Sensitivity analyses supported these findings (Table 3; eTable 10 and 11 in Supplement 1). Multivariable analyses using matching or IPW found a significant association between AI use and radiologist burnout. This association remained robust when substituting overall workload and AI acceptance with individual factors. The association pattern persisted across different regions, even though significance was not consistently achieved.

## Discussion

To our knowledge, this study is the first to investigate the association between AI use and radiologist burnout using a large, nationwide cross-sectional sample. We found that AI use was associated with increased odds of burnout among radiologists, exhibiting a significant dose-response association. Moreover, joint exposure to AI use alongside either high workload or low AI acceptance was associated with an additional risk of burnout. These results underscore the need to reassess the role of AI technology in mitigating radiologist burnout. Balancing AI use with an appropriate radiology workforce and maintaining psychological acceptance of AI technology in clinical practice is essential.

The increased burnout associated with AI use in our study primarily stems from EE, driven by elevated work demands and overload.^1^ Previous studies have identified work overload as a principal risk factor for physician burnout.^3,4^ To address workload issues in radiology, there is growing interest in AI-assisted imaging identification. Prior studies suggest that AI use could reduce radiologists’ workload by 40% to 90% in cancer screening by minimizing repetitive tasks and unnecessary discussions.^25,26^ However, the potential drawbacks of AI technologies in clinical settings have often been overlooked. Recent discussions have examined the divergent impacts of AI in public health screening vs clinical care.^27^ Unlike in screening, where high sensitivity of AI may reduce workload, the clinical setting often necessitates more radiologist time for differential diagnoses. For example, researchers found that AI could decrease reading times for radiologists when no abnormalities are detected, but it could increase reading times when abnormalities are present.^28^ Furthermore, the increased workload attributed to AI often results from elevated postprocessing and interpretation times.^16^ Our study underscores a critical knowledge gap, demonstrating a positive association between AI use and radiologist burnout, which longitudinal studies should further explore.

Our results indicate that the association of AI use with burnout may be exacerbated by increasing workload. The integration of AI technology in hospitals could lead to higher consultation volumes and increase radiologists’ workload. Although data to substantiate this are limited, policymakers are considering increasing radiology capacity with AI to address rising care demands, particularly in tertiary hospitals.^29,30^ For radiologists, the inherently isolated and sedentary nature of their work contributes to higher burnout rates compared with other specialties.^4,31^ AI may further exacerbate these challenges by diminishing opportunities for peer collaboration and patient interaction, while fears of job displacement and uncertainties surrounding AI use heighten stress.^32,33,34^ Joint association analyses showed that the risk of burnout associated with AI use was particularly pronounced among radiologists with high workloads, highlighting the urgent need to explore effective coordination strategies between radiologists and AI.

Existing evidence links physician pressure to increase knowledge and skills in clinical practice and job satisfaction to burnout.^4,31^ Those lacking clinical experience, such as junior or younger physicians, as well as those reporting low job satisfaction, are at a heightened risk for burnout. Our study found a joint association of AI use and high AI acceptance on radiologist burnout. The integration of AI in radiology practice necessitates a shift in knowledge structure, requiring radiologists to learn how to operate, interpret, and interact with these technologies. Radiologists with negative attitudes toward AI, limited knowledge, or a lower intention to use AI may experience diminished job satisfaction, particularly when required to implement AI in their practice. To our knowledge, no prior study has examined modifiable factors of the association between between AI use and burnout. Although there are growing concerns that AI may alleviate physician workload across various medical specialties, the harmonizing of the relationship between physicians and AI tools has not yet been appropriately prioritized. Our findings provide insights in this regard.

A strength of our study is the representative sample of radiologists selected through a national radiology quality control system, ensuring a higher response rate compared with other studies.^3,10,32^ Participants were enrolled from approximately 70% of prefecture-level cities in all 31 provinces of China, representing a wide range of personal and professional characteristics. Our sample size is the largest among recent studies, enabling analyses with sufficient statistical power.

Radiologists, already in short supply, are overwhelmed by rapidly growing health care needs and medical imaging data.^35,36^ In China, the annual growth rate of medical imaging data are 7.5 times that of radiologists.^30^ Therefore, radiologists’ workload and burnout are receiving unprecedented attention. AI technology offers a potential solution to the shortage of radiologists. Policymakers and researchers are planning or have implemented AI strategies in radiology to address the supply-demand imbalance while maintaining the same or a slowly growing radiology workforce. However, the excitement and expectations surrounding technological advances should not overshadow the challenges that remain before AI can be routinely applied in radiology practice. AI tools must provide clinical results that radiologists can understand and trust to truly reduce workload. Few AI technologies have been rigorously validated in randomized clinical trials.^37^ Furthermore, integrating AI tools into the radiology workflow should be a key research task.^29,38^ Although further work is needed to establish causal relationships and intervention feasibility, our findings emphasize the importance of creating a suitable environment for the interaction between radiologist and AI. This includes improving the interpretability of AI tools, providing education about AI technology, and increasing AI acceptance among radiologists.

### Limitations

Our study has limitations. Despite an approximately 90% response rate, there is a possibility that nonrespondents may be too burned out to participate, potentially introducing nonresponse bias. Including only secondary and higher hospitals may result in insufficient representation of primary hospitals. The cross-sectional design also complicates the ability to confirm the direction of the association. While we suggest that AI use is a risk factor for burnout, it is plausible that burnout could drive the adoption of AI in practice. Additionally, we could not ascertain whether the effects would diminish as radiologists become more adept with AI. Nevertheless, our stratified and dose-response analyses strengthen the validity of our findings. Moreover, we did not collect data on leadership within radiology practices, a factor known to influence burnout.^39,40^ Additionally, the frequency of AI use was determined through self-report rather than objective records, leaving room for measurement error.

## Conclusions

In this cross-sectional study of radiologist burnout, AI use was associated with higher odds of radiologist burnout, with a significant dose-response association. The association was moderated by radiologists’ workload and AI acceptance. The role of AI in alleviating radiologist burnout should be considered cautiously, and longitudinal studies are warranted to further elucidate this association.

## Notes

Supplement 1. **eAppendix 1.** Description of the Sampling Design in the Survey **eAppendix 2.** Assessment of Workload and AI Acceptance **eAppendix 3.** Gallup’s Employee Engagement Scale **eAppendix 4.** Statistical Analysis **eFigure 1.** Flowchart of Study Cohort **eFigure 2.** Association of AI Use With Burnout and Its Components by Subgroups **eFigure 3.** Predicted Probability of Burnout for Joint Exposure of AI Use, Workload, and AI Acceptance **eTable 1.** Distribution of Participants and Response Rate **eTable 2.** Personal and Professional Characteristics Between Included and Excluded Radiologists **eTable 3.** Personal and Professional Characteristics after Propensity Score Matching **eTable 4.** Weighted Prevalence of Radiologist Burnout Between the AI and Non-AI Groups **eTable 5.** Dose-Response Association Between the Frequency of AI Use and Burnout in Radiology Practice by Subgroups **eTable 6.** Dose-Response Association Between the Frequency of AI Use and Emotional Exhaustion in Radiology Practice by Subgroups **eTable 7.** Dose-Response Association Between the Frequency of AI Use and Depersonalization in Radiology Practice by Subgroups **eTable 8.** Joint Association of AI Use, Workload, and AI Acceptance With Emotional Exhaustion **eTable 9.** Joint Association of AI Use, Workload, and AI Acceptance With Depersonalization **eTable 10.** Association of AI Use With Burnout Adjusting for Workload Factors in Regression Models **eTable 11.** Association of AI Use With Burnout by Geographic Locations

Supplement 2.
Data Sharing Statement
