# Supplementary material for: Artificial Intelligence and Radiologist Burnout
Source: JAMA Netw Open. 2024 Nov 22;7(11):e2448714. doi: 10.1001/jamanetworkopen.2024.48714 (PMC11584928; doi:10.1001/jamanetworkopen.2024.48714)
Supplement: Supplement 1. — eAppendix 1. Description of the Sampling Design in the Survey eAppendix 2. Assessment of Workload and AI Acceptance eAppendix 3. Gallup’s Employee Engagement Scale eAppendix 4. Statistical Analysis eFigure 1. Flowchart of Study Cohort eFigure 2. Association of AI Use With Burnout and Its Components by Subgroups eFigure 3. Predicted Probability of Burnout for Joint Exposure of AI Use, Workload, and AI Acceptance eTable 1. Distribution of Participants and Response Rate eTable 2. Personal and Professional Characteristics Between Included and Excluded Radiologists eTable 3. Personal and Professional Characteristics after Propensity Score Matching eTable 4. Weighted Prevalence of Radiologist Burnout Between the AI and Non-AI Groups eTable 5. Dose-Response Association Between the Frequency of AI Use and Burnout in Radiology Practice by Subgroups eTable 6. Dose-Response Association Between the Frequency of AI Use and Emotional Exhaustion in Radiology Practice by Subgroups eTable 7. Dose-Response Association Between the Frequency of AI Use and Depersonalization in Radiology Practice by Subgroups eTable 8. Joint Association of AI Use, Workload, and AI Acceptance With Emotional Exhaustion eTable 9. Joint Association of AI Use, Workload, and AI Acceptance With Depersonalization eTable 10. Association of AI Use With Burnout Adjusting for Workload Factors in Regression Models eTable 11. Association of AI Use With Burnout by Geographic Locations [file jamanetwopen-e2448714-s001.pdf]

Supplemental Online Content

Liu H, Ding N, Li X, et al. Artificial intelligence and radiologist burnout. *JAMA Netw Open.* 2024;7(11):e2448714. doi:10.1001/jamanetworkopen.2024.48714

eAppendix 1. Description of the Sampling Design in the Survey

eAppendix 2. Assessment of Workload and AI Acceptance

eAppendix 3. Gallup's Employee Engagement Scale

eAppendix 4. Statistical Analysis

eFigure 1. Flowchart of Study Cohort

eFigure 2. Association of AI Use With Burnout and Its Components by Subgroups

eFigure 3. Predicted Probability of Burnout for Joint Exposure of AI Use, Workload, and AI Acceptance

eTable 1. Distribution of Participants and Response Rate

eTable 2. Personal and Professional Characteristics Between Included and Excluded Radiologists

eTable 3. Personal and Professional Characteristics after Propensity Score Matching.

eTable 4. Weighted Prevalence of Radiologist Burnout Between the AI and Non-AI Groups

eTable 5. Dose-Response Association Between the Frequency of AI Use and Burnout in Radiology Practice by Subgroups

eTable 6. Dose-Response Association Between the Frequency of AI Use and Emotional Exhaustion in Radiology Practice by Subgroups

eTable 7. Dose-Response Association Between the Frequency of AI Use and Depersonalization in Radiology Practice by Subgroups

eTable 8. Joint Association of AI Use, Workload, and AI Acceptance With Emotional Exhaustion

eTable 9. Joint Association of AI Use, Workload, and AI Acceptance With Depersonalization

eTable 10. Association of AI Use With Burnout Adjusting for Workload Factors in Regression Models

eTable 11. Association of AI Use With Burnout by Geographic Locations

This supplemental material has been provided by the authors to give readers additional information about their work.

© 2024 Liu H et al. *JAMA Network Open.*

eAppendix 1. Description of the sampling design in the survey.

The sampling design for this survey is based on the the National Center for Quality Control of Radiology (NCQCR), which was established to improve healthcare quality in China. In 2023, China initiated the "National Action Plan for Comprehensive Improvement of Medical Quality (2023-2025)," which aims to enhance the quality of medical services in secondary and tertiary hospitals [1]. As part of this initiative, the National Health Commission of the People's Republic of China announced the first batch of national quality control centers, which includes the specialty of radiology [2]. These centers serve as critical infrastructures for maintaining and improving medical standards, which has been reported by other specialty [3].

The NCQCR operates as a tiered system, comprising four levels: national, provincial, municipal, and county. At the national level, Peking Union Medical College Hospital is designated as the primary center. Each province then selects one tertiary hospital as the provincial quality control center, while each municipality selects a public, secondary or higher-level hospital to serve as the municipal center. Lastly, each county selects a secondary or higher-level hospital as the county-level quality control center. Furthermore, the NCQCR uses a sentinel surveillance approach to establish a regular monitoring system, and sentinel sites are selected based on several criteria as follows: (1) each provincial center selects 10 to 15 hospitals; (2) the hospital must be at least a secondary-level facility; (3) hospitals in tier network or specialized hospitals (e.g., for women and children, cancer, and cardiovascular diseases) should be chosen, with one of each type being selected; (4) hospitals that have demonstrated a strong ability to collaborate on quality control efforts are prioritized. Finally, 392 sentinel hospitals have been established, covering all 31 provinces and the Xinjiang Production and Construction Corps in mainland China. These hospitals serve as the foundation for the quality control network, continuously monitoring radiological practices across the country (**eFigure A1**).

In 2023, there are 3,855 tertiary hospitals and 11,946 secondary hospitals in China [4]. In this study, we selected 392 sentinel hospitals and 751 quality control hospitals at the municipal and county levels that were already established within the four-tier network, bringing the total to 1,143 hospitals. The overall sample size of the survey reached 7.2% of the total number of 2/3 hospitals in mainland China. Radiologists from each hospital were selected according to the following inclusion criteria: (1) 1 to 5 radiologists were chosen per hospital for study participation; (2) participants had a minimum of one year of professional experience because radiologists always need to have at least one year of rotation training as well as psychological adjustments [5-7]; (3) participants joined the hospital below the legal retirement age of 60 because those joining the hospital after the age of 60 have greater flexibility in their working roles; (4) participants were not pregnant at the time of the survey; and (5) participation was voluntary, with all responses being completed through an anonymous survey. The study was approved by the Ethics Committee of the Peking Union Medical College Hospital (No.S-K1538).

© 2024 Liu H et al. *JAMA Network Open.*

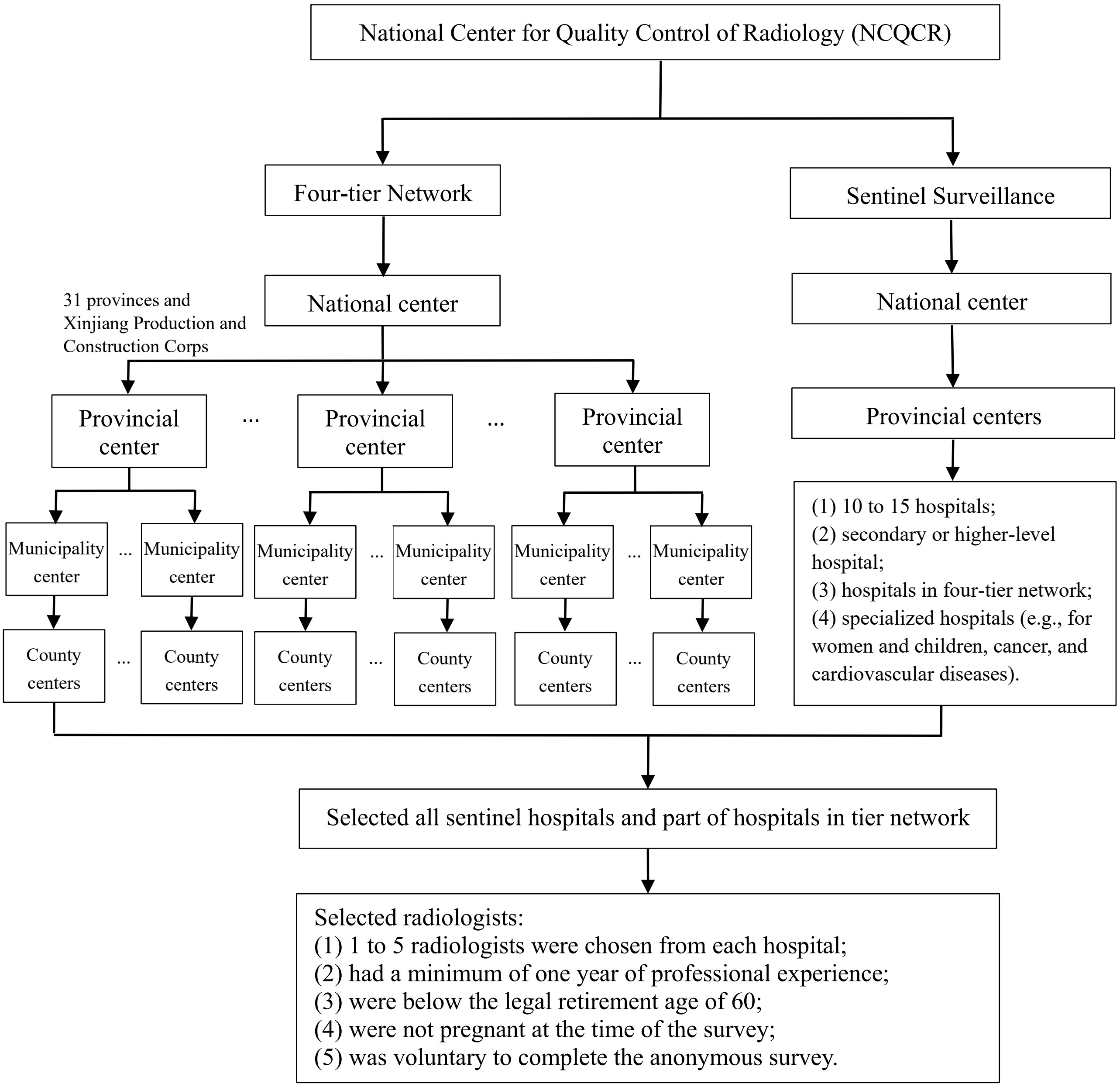

eFigure A1. Sampling design in this survey.

Reference:

[1] National Health Commission of the People's Republic of China. Implementing the Action Plan for Comprehensive Improvement of Medical Quality (2023-2025) [In Chinese]. https://www.gov.cn/zhengce/zhengceku/202305/content_6883704.htm. May 26, 2023.

[2] National Health Commission of the People's Republic of China. Administrative Regulations for Medical Quality Control Centers [In Chinese]. https://www.gov.cn/zhengce/zhengceku/2023-02/28/content_5743657.htm. February 22, 2023.

[3] Gao L, Li J, Gu Y, Ma L, Xu W, Tao X, et al. Breast ultrasound in Chinese hospitals: A cross-sectional study of the current status and influencing factors of BI-RADS utilization and diagnostic accuracy. *Lancet Reg Health West Pac*. 2022;29:100576.

[4] National Health Commission of the People's Republic of China. 2023 Statistical Bulletin on

© 2024 Liu H et al. *JAMA Network Open.*

the Development of China's Health and Medical Services [In Chinese]. http://www.nhc.gov.cn/guihuaxxs/s3585u/202408/6c037610b3a54f6c8535c515844fae96/files/58c5d1e9876344e5b1aa5aa2b083a51a.pdf. August 26, 2024.
[5] Zhang J, Han X, Yang Z, Wang Z, Zheng J, Yang Z, Zhu J. Radiology residency training in China: results from the first retrospective nationwide survey. Insights Imaging. 2021;12(1):25.
[6] Chinese Medical Doctor Association. Contents and Standards of the National Standardized Residency Training [in Chinese]. Available at: https://www.ccgme-cmda.cn/news/15117/1/article. August 5, 2022.
[7] Nguyen MT, Henrichsen T, Bhatt A. Exploring your Interest in Radiology: A First-Year Medical Student's Perspective. Curr Probl Diagn Radiol. 2022;51(6):813-814.

© 2024 Liu H et al. *JAMA Network Open.*

eAppendix 2. Assessment of Workload and AI Acceptance.

1. Assessment of AI acceptance

According to the Technology Acceptance Model, the acceptance of a new technology was determined by perceived usefulness and perceived ease of use. Use of new technology was directly driven by behavioral intention, which was affected by attitude toward use and perceived usefulness. Therefore, we used AI related knowledge, confidence, attitude, and intention to construct an overall AI acceptance. In our study, we assessed AI-related psychological factors by asking "o you have any knowledge of computer science, computer engineering, or statistics? Yes and I had qualification, Yes but I did not have qualification, No.", "What do you think is the future of AI in radiology medicine? Very optimistic and AI may reduce radiologist's workload, very optimistic but fear that AI will replace radiologists, neutrality and AI would not affect the medical field in short time, Pessimistic and AI would have little impact on the medical field.", "Do you agree that AI could reduce the workload in radiology practice? Agree or disagree.", and "Do you plan to take the initiative to learn AI-related knowledge or skills? Yes, no, or Not sure". In order to have enough samples to converge the model, we reclassified these variables into two groups (low and high), considering self-reported classification and sample size within these levels. Then, we constructed AI acceptance with latent class analysis, using R poLCA package.

We fitted several models with two through four latent classes and all models converged. Considering the interpretation of latent classes in our analysis, we selected the two-latent class solution. In the two-latent class solution, the AIC is 27530.8, BIC is 27592.1, G2 is 193.4, and mean posterior probabilities of all latent classes were above 0.70. As shown in below table, latent class 1 was characterized by low-level knowledge and confidence, relatively high attitude, and relatively low intention, which could be defined as "low AI acceptance"; latent class 2 was characterized by high-level knowledge, confidence, attitude, and intention about AI use in radiology, which could be defined as "high AI acceptance".

Supplementary table. Characteristics of different latent class solutions.

	**Latent class**				**AIC**	**BIC**	**G^2**
	**Class 1**	**Class 2**	**Class 3**	**Class 4**			
**Two-latent class solution**					27530.8	27592.12	193.4
Prevalence	0.22	0.78	NA	NA			
Knowledge low	0.73	0.47	NA	NA			
Knowledge high	0.27	0.53	NA	NA			
Confidence low	0.60	0.22	NA	NA			
Confidence high	0.40	0.78	NA	NA			
Attitude low	0.21	0.01	NA	NA			
Attitude high	0.79	0.99	NA	NA			
Intention low	0.58	0.06	NA	NA			
Intention high	0.42	0.93	NA	NA			
**Three-latent class solution**					27355.1	27450.5	7.71
Prevalence	0.19	0.05	0.76	NA			

© 2024 Liu H et al. *JAMA Network Open.*

Knowledge low	0.80	0.57	0.48	NA			
Knowledge high	0.20	0.43	0.51	NA			
Confidence low	0.44	0.99	0.25	NA			
Confidence high	0.56	0.01	0.75	NA			
Attitude low	0.07	0.67	0.02	NA			
Attitude high	0.93	0.33	0.98	NA			
Intention low	0.90	0.55	0.03	NA			
Intention high	0.10	0.45	0.97	NA			
**Four-latent class solution**					27357.4	27486.8	0.0045
Prevalence	0.06	0.17	0.75	0.02			
Knowledge low	0.47	0.03	0.01	0.30			
Knowledge high	0.53	0.97	0.99	0.70			
Confidence low	0.90	0.28	0.23	0.84			
Confidence high	0.10	0.72	0.77	0.16			
Attitude low	0.43	0.03	0.01	0.30			
Attitude high	0.57	0.97	0.99	0.70			
Intention low	0.44	0.38	0.03	0.99			
Intention high	0.56	0.62	0.97	0.01			

2. Assessment of workload

Because multiple workload factors were interrelated and reflecting different meanings, the overall workload was conducted based on five workload factors, including working hours spent on image interpretation per week, the amount of image interpretation per day, the main device type used in work, the role in the reporting workflow, and hospital level. According to the median of the sample, working hours was categorized as <42 hours and ≥42 hours per week, and the amount of image interpretation was categorized as <120 and ≥120 per day. The main device type was defined as that radiologist read the most mount of images per day according to radiologists self report. According to the difficulty of image interpretation, the device type was classified as CT or MRI and X-ray. The role in the reporting workflow was categorized as initial or second opinion and both work. The former group represents low workload and the later represents high workload.

For each factor, we assigned 0 point for low workload and 1 point for high workload. The overall workload score was the sum of the points ranging from 0 to 5, with higher scores indicating high workload. We categorized radiologists into three class: low (0-2 point), medium (3 points), and high (4-5 points).

© 2024 Liu H et al. *JAMA Network Open.*

eAppendix 3. Gallup's Employee Engagement Scale.

Q01	I know what is expected of me at work.
Q02	I know what is expected of me at work.
Q03	At work, I have the opportunity to do what I do best every day.
Q04	In the last seven days, I have received recognition or praise for doing good work.
Q05	My supervisor, or someone at work, seems to care about me as a person.
Q06	There is someone at work who encourages my development.
Q07	At work, my opinions seem to count.
Q08	The mission or purpose of my company makes me feel my job is important.
Q09	My associates or fellow employees are committed to doing quality work.
Q10	I have a best friend at work.
Q11	In the last six months, someone at work has talked to me about my progress.
Q12	This last year, I have had opportunities at work to learn and grow.

In the scale, Q3 measures the perception of control in working, Q4 measures the perception of spiritual rewards, Q8 measures the perception of values associated with work, Q5 measures the perception of support from organization, and Q9 measures the perception of support from coworkers. All questions are measured using Likert scale from very agree to very disagree. We classified them into two class: agree (strongly agree or agree) and disagree (neutral, disagree, or strongly disagree).

© 2024 Liu H et al. *JAMA Network Open.*

eAppendix 4. Statistical Analysis.

1. Propensity score calculation

In order to adjust for confounding between the AI and non-AI groups, a propensity score-based analysis strategy was developed. Propensity scores were calculated for individuals in each group using a logistic regression model with the AI group as the dependent variable, adjusted for individual and professional characteristics, workload, AI acceptance, and psychological factors of job satisfaction. For each individual, a predicted probability of belonging to the AI group was calculated, and the propensity score was defined as the inverse of the predicted probability.

2. Propensity score-based matching

Individual matching represents an alternative approach to improving the balance of covariates between the AI and non-AI groups. In this study, radiologists in the AI group were matched 1:1 to the comparison group based on propensity scores. The two groups were matched using the caliper matching algorithm with a caliper value of 0.1 standard deviations. In essence, an individual in the AI group would be matched to a comparison in the non-AI group based on the difference in propensity score, which was lower than the caliper value. In the event that more than one individual met the stipulated criteria, a random allocation process was employed. The proposed methodology would ensure an equitable distribution of covariates between the two groups. Nevertheless, it is possible that some individuals may be excluded from the study due to failure to pair.

3. Generalized linear regression model with inverse probability weighting

We fitted generalized linear regressions to examine the association between AI use and radiologist burnout, adjusting for personal and professional characteristics, workload score, AI acceptance, and psychological factors of job satisfaction. Based on the propensity score and matching, we developed three model: (1) a multivariable model with inverse probability weighting, in which the model was weighted using propensity score; (2) a multivariable model based on matched samples, in which the model was fitted among samples created through propensity score matching using the nearest neighbor method to establish a control group; (3) a multivariable model incorporating the propensity score as an additional covariate. We regarded results from the model (1) as the main analysis and others as sensitivity analyses.

Given that our outcome variable is binary, we employed a logistic regression model. To account for the multistage clustered sampling process, we incorporated random effects at province level to adjust for differences among hospitals in various regions. Due to the non-integer nature of the estimated probability weights, we fitted a negative binomial distribution to accommodate overdispersion [1]. The regression equations were fitted using the glmmTMB package in R software to estimate odds ratios (ORs) reflecting the strength of associations. Notably, with a burnout rate exceeding 10%, the ORs may not accurately represent prevalence differences between groups; thus, we further fitted a binomial-log regression model to calculate prevalence ratios (PRs) [2]. Based on the PRs, we estimated additive interactions (relative excess risk due to interaction, RERI) and multiplicative interactions (relative prevalence due to interaction, RPR), employing the Hosmer method to estimate confidence intervals for RERI [3].

© 2024 Liu H et al. *JAMA Network Open.*

Reference:

[1] Coxe S, West SG, Aiken LS. The analysis of count data: a gentle introduction to poisson regression and its alternatives. *J Pers Assess*. 2009;91(2):121-136.

[2] Petersen MR, Deddens JA. A comparison of two methods for estimating prevalence ratios. *BMC Med Res Methodol*. 2008;8:9.

[3] Hosmer DW, Lemeshow S. Confidence interval estimation of interaction. *Epidemiology*. 1992;3(5):452-456.

© 2024 Liu H et al. *JAMA Network Open.*

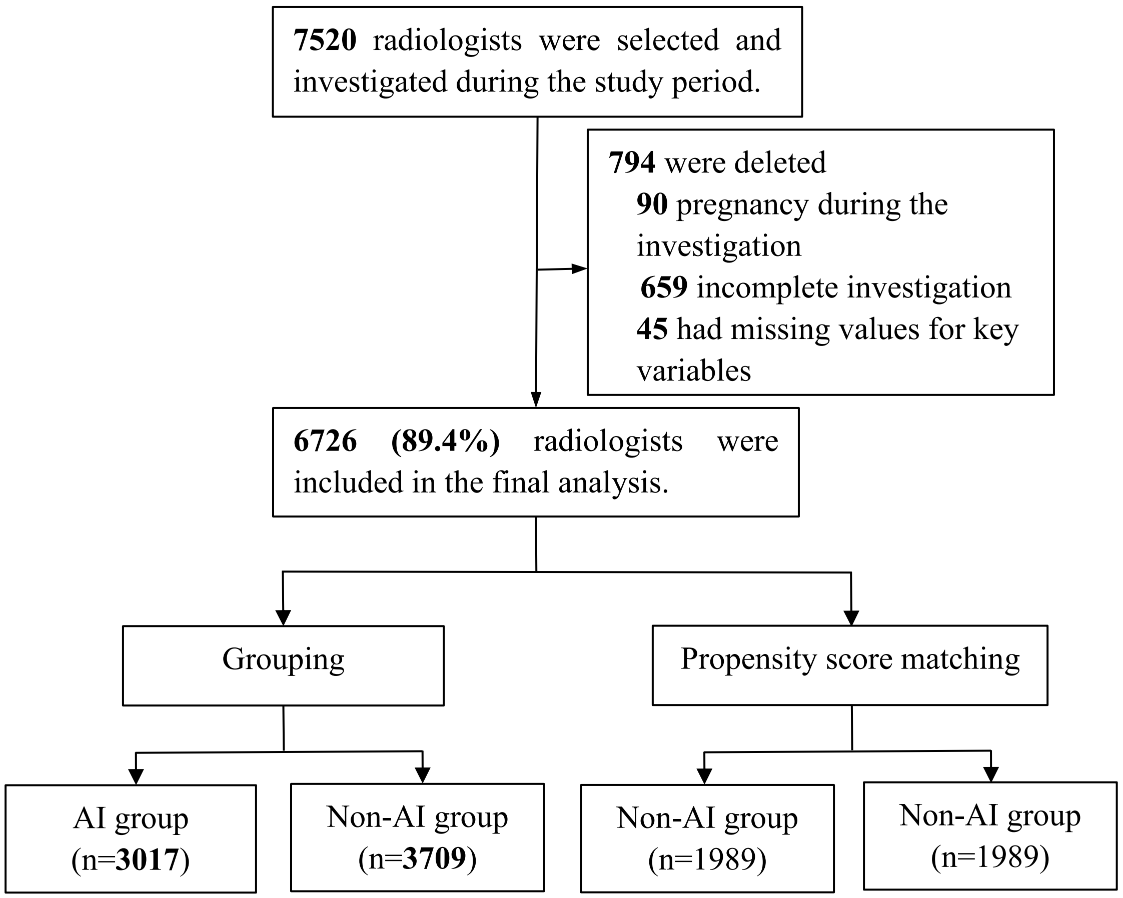

eFigure 1. Flowchart of Study Cohort

AI=artificial intelligence.

© 2024 Liu H et al. *JAMA Network Open.*

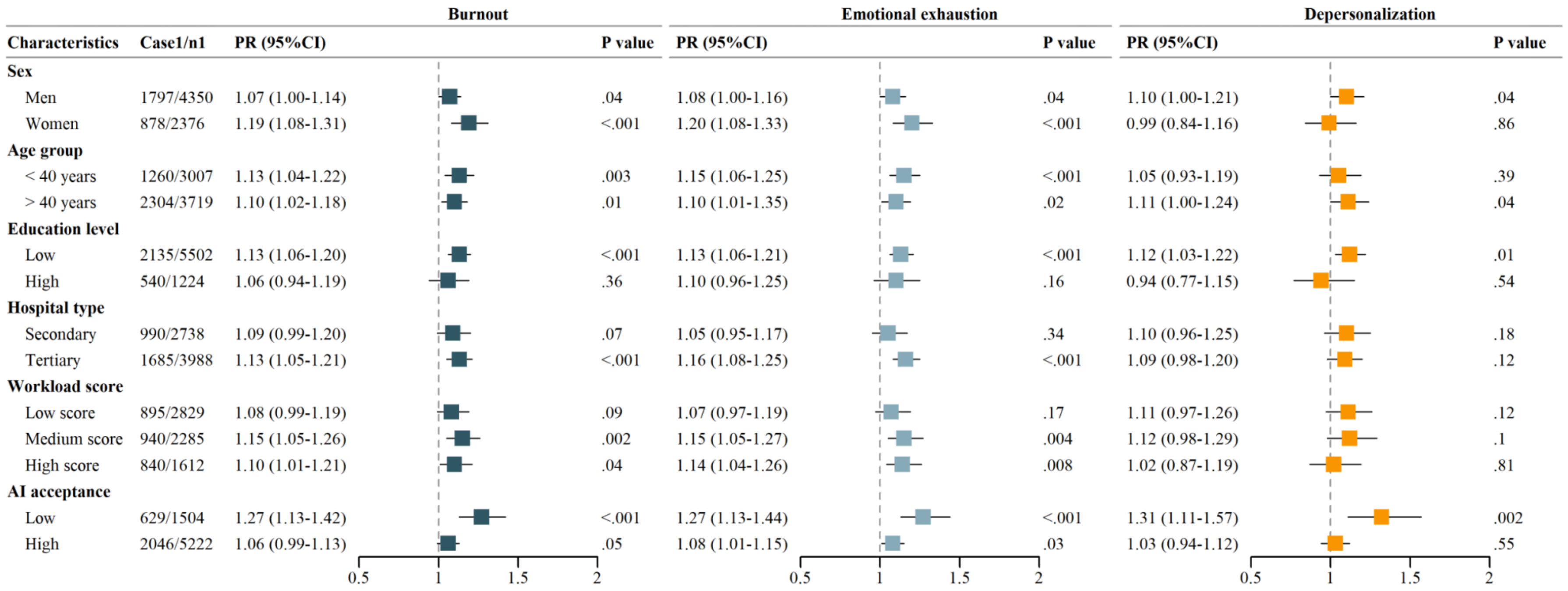

Characteristics	Case1/n1	Burnout PR (95%CI)	Burnout P value	Emotional exhaustion PR (95%CI)	Emotional exhaustion P value	Depersonalization PR (95%CI)	Depersonalization P value
**Sex**							
Men	1797/4350	1.07 (1.00-1.14)	.04	1.08 (1.00-1.16)	.04	1.10 (1.00-1.21)	.04
Women	878/2376	1.19 (1.08-1.31)	<.001	1.20 (1.08-1.33)	<.001	0.99 (0.84-1.16)	.86
**Age group**							
< 40 years	1260/3007	1.13 (1.04-1.22)	.003	1.15 (1.06-1.25)	<.001	1.05 (0.93-1.19)	.39
> 40 years	2304/3719	1.10 (1.02-1.18)	.01	1.10 (1.01-1.35)	.02	1.11 (1.00-1.24)	.04
**Education level**							
Low	2135/5502	1.13 (1.06-1.20)	<.001	1.13 (1.06-1.21)	<.001	1.12 (1.03-1.22)	.01
High	540/1224	1.06 (0.94-1.19)	.36	1.10 (0.96-1.25)	.16	0.94 (0.77-1.15)	.54
**Hospital type**							
Secondary	990/2738	1.09 (0.99-1.20)	.07	1.05 (0.95-1.17)	.34	1.10 (0.96-1.25)	.18
Tertiary	1685/3988	1.13 (1.05-1.21)	<.001	1.16 (1.08-1.25)	<.001	1.09 (0.98-1.20)	.12
**Workload score**							
Low score	895/2829	1.08 (0.99-1.19)	.09	1.07 (0.97-1.19)	.17	1.11 (0.97-1.26)	.12
Medium score	940/2285	1.15 (1.05-1.26)	.002	1.15 (1.05-1.27)	.004	1.12 (0.98-1.29)	.1
High score	840/1612	1.10 (1.01-1.21)	.04	1.14 (1.04-1.26)	.008	1.02 (0.87-1.19)	.81
**AI acceptance**							
Low	629/1504	1.27 (1.13-1.42)	<.001	1.27 (1.13-1.44)	<.001	1.31 (1.11-1.57)	.002
High	2046/5222	1.06 (0.99-1.13)	.05	1.08 (1.01-1.15)	.03	1.03 (0.94-1.12)	.55

eFigure 2. Association of AI Use with Burnout and its Components by Subgroups

Burnout was defined as having at least one symptom of the emotional exhaustion (≥27) or depersonalization (≥10). The square point represents PRs, and the bar represents 95%CI. PRs calculated for the joint exposure of AI use and workload score groups by binomial-log regression, adjusting for individual and professional characteristics and psychological factors. All analyses were calculated using inverse propensity weighting.

AI=artificial intelligence. PR=prevalence ratio. CI=confidence interval.

© 2024 Liu H et al. *JAMA Network Open.*

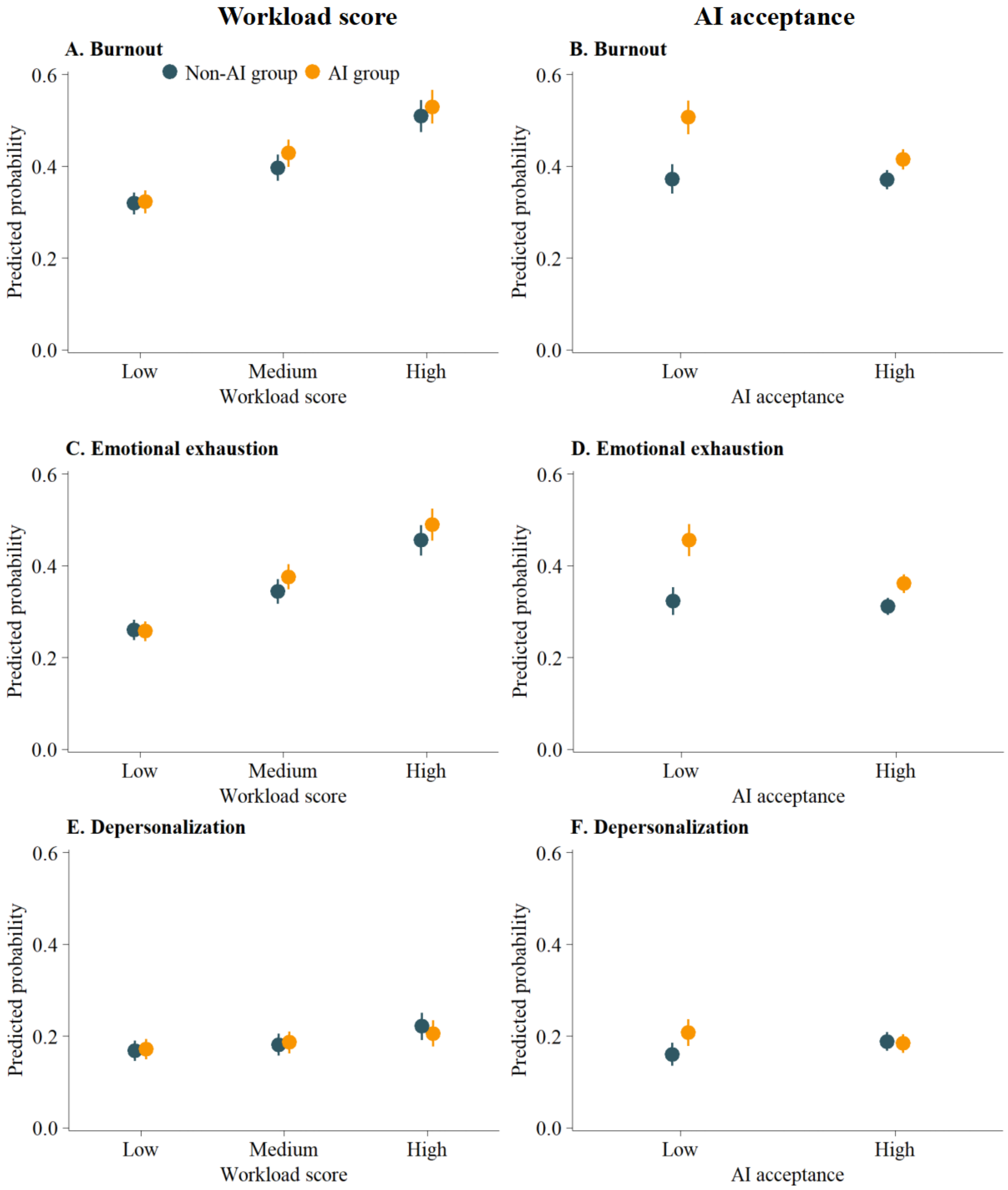

eFigure 3. Predicted probability of Burnout for Joint Exposure of AI use, Workload, and AI Acceptance.

Predicted probabilities of burnout were estimated by the generalized linear regression models with IPW and plotted by marginaleffects package in R statistical software. A interaction term of AI use and workload score or AI acceptance was included in the models.
AI=artificial intelligence. CI=confidence interval.

© 2024 Liu H et al. *JAMA Network Open.*

eTable 1. Distribution of Participants and Response Rate

Province	Number of hospitals	Participants	Eligible Participants	Response rate (%)
Anhui	36	178	158	88.8
Beijing	20	135	119	88.1
Chongqing	17	118	105	89.0
Fujian	46	299	282	94.3
Gansu	34	201	178	88.6
Guangdong	42	231	197	85.3
Guangxi	54	328	300	91.5
Guizhou	49	315	280	88.9
Hainan	29	130	115	88.5
Hebei	134	902	821	91.0
Heilongjiang	15	66	58	87.9
Henan	15	54	46	85.2
Hubei	82	519	460	88.6
Hunan	17	63	56	88.9
Inter Mongolia	16	78	69	88.5
Jiangsu	45	281	255	90.7
Jiangxi	23	127	113	89.0
Jilin	59	379	336	88.7
Liaoning	78	495	436	88.1
Ningxia	11	59	52	88.1
Qinghai	14	75	67	89.3
Shandong	110	711	626	88.0
Shanghai	3	13	12	92.3
Shannxi	2	8	7	87.5
Shanxi	56	350	314	89.7
Sichuan	77	474	437	92.2
Tianjin	18	123	112	91.1
Xinjiang	38	202	180	89.1
Xizang	8	29	23	79.3
Yunnan	67	420	365	86.9
Zhejiang	28	157	147	93.6

© 2024 Liu H et al. *JAMA Network Open.*

eTable 2. Personal and Professional Characteristics between Included and Excluded Radiologists

	Included (n=6726)	Excluded (n=794)	*P* value
**Personal characteristics**			
Age (years)	41.0 (34.0, 48.0)	33.0 (28.0, 41.0)	<.001
Female	2376 (35.3)	298 (37.5)	.22
Having children	5493 (81.7)	491 (69.7)	<.001
Geographic region			.37
Eastern	2686 (39.9)	295 (37.2)	
Central	1147 (17.1)	144 (18.2)	
Western	2063 (30.7)	110 (13.9)	
Northeast	830 (12.3)	244 (30.8)	
Relationship status			<.001
Single	905 (13.5)	164 (20.7)	
Married	5716 (85.0)	609 (76.7)	
Others [a]	105 (1.5)	21 (2.6)	
Monthly income, <5000	5447 (80.9)	684 (86.2)	<.001
Education levels, low	5502 (81.8)	633 (79.7)	.15
**Professional characteristics**			
AI use in practice	3017 (44.9)	291 (36.7)	<.001
Specialty, breast, chest, or blood vessel	5513 (82.0)	576 (72.5)	<.001
Years in practice (years)	16.0 (8.0, 25.0)	10.0 (5.0, 21.0)	<.001
Hours worked per week (hours)	42.0 (40.0, 50.0)	40.0 (35.0, 50.0)	<.001
Numbers of images per day	115 (72, 180)	120 (77, 197)	.01
General hospital	6066 (90.2)	703 (88.5)	.14
Senior professional title	2500 (37.2)	191 (24.1)	<.001
Main working in practice			<.001
X-ray	2526 (37.6)	333 (41.9)	
Computerized tomography	3754 (55.8)	378 (47.6)	
MRI	446 (6.6)	83 (10.5)	
Role in the workflow			<.001
First opinion	1774 (26.4)	360 (45.3)	
Second opinion	1738 (25.8)	136 (17.1)	
Both	3214 (47.8)	298 (37.5)	
Attitude toward AI, positive	6253 (93.0)	713 (89.8)	.001
Knowledge on AI, familiar	3047 (45.3)	432 (54.4)	<.001

Data are represented as n (%) or median (inter-quarter range). [a] Others include divorce, separation, widowed or widower. AI=artificial intelligence.

© 2024 Liu H et al. *JAMA Network Open.*

eTable 3. Personal and Professional Characteristics after Propensity Score Matching

	Propensity score matching	
	**AI group (n=1989)**	**No AI group (n=1989)**
**Personal characteristics**		
Age (years)	40 (34, 48)	40 (34, 48)
Female	723 (36.4)	717 (36.1)
Male	1266 (63.6)	1272 (63.9)
Having children	1622 (81.6)	1635 (82.2)
Geographic region		
Eastern	893 (44.9)	819 (41.2)
Central	337 (16.9)	326 (16.4)
Western	507 (25.5)	600 (30.2)
Northeast	252 (12.7)	244 (12.3)
Relationship status		
Single	265 (13.3)	268 (13.5)
Married	1695 (85.2)	1682 (84.6)
Others [a]	29 (1.5)	39 (2.0)
Monthly income, <5000	1624 (81.7)	1637 (82.3)
Education levels, low	1630 (82.0)	1679 (84.4)
**Professional characteristics**		
Specialty, breast, chest, or blood vessel	1671 (84.0)	1617 (81.3)
Years in practice (years)	16.0 (8.0-25.0)	16.0 (9.0-25.0)
Hours worked per week (hours)	43.5 (40.0-50.0)	42.0 (40.0-50.0)
Numbers of images per day	123 (80-193)	120 (80-189)
General hospital	1868 (93.9)	1755 (88.2)
Senior professional title	1186 (59.6)	1250 (62.9)
Main working in practice		
X-ray	729 (36.7)	662 (33.3)
Computerized tomography	1133 (57.0)	1193 (60.0)
MRI	127 (6.4)	134 (6.4)
Role in practice		
Initial report	497 (25.0)	543 (27.3)
Final report	593 (29.8)	482 (24.2)
Both	899 (45.2)	964 (48.5)
Attitude toward AI, positive	1923 (96.7)	1792 (90.1)
Knowledge on AI, familiar	970 (48.8)	876 (44.0)

© 2024 Liu H et al. *JAMA Network Open.*

eTable 4. Weighted Prevalence of Radiologist Burnout between the AI and Non-AI Groups

	**AI group**	**Non-AI group**	**P**
Sex			
Male	43.8 (41.3-36.4)	41.4 (39.1-43.7)	.16
Female	39.9 (36.6-43.1)	33.2 (30.1-36.3)	.004
Age group			
<40 years	44.6 (41.8-47.5)	40.1 (37.1-43.1)	.03
≥40 years	40.6 (37.8-43.4)	37.2 (34.9-35.9)	.06
Education level [a]			
Low	41.9 (39.5-44.2)	36.9 (35.2-38.6)	<.001
High	44.9 (41.6-48.3)	45.1 (38.8-51.5)	.96
Monthly income			
<5000	42.9 (40.6-45.3)	38.0 (36.2-39.7)	<.001
≥5000	40.6 (34.6-46.6)	40.2 (36.8-43.5)	.91
Workload score			
Low score	33.3 (29.8-36.9)	31.3 (29.2-33.3)	.32
Medium score	44.7 (41.6-47.8)	38.7 (35.5-41.9)	.008
High score	54.6 (51.4-57.9)	50.3 (45.5-54.9)	.13
AI acceptance			
Low	49.7 (44.3-55.0)	37.4 (34.4-40.5)	<.001
High	40.4 (38.3-42.4)	38.8 (36.6-41.0)	.30

Data are represented as prevalence with 95%CI. The prevalence was estimated using inverse probability weighting and 95%CI was estimated by bootstrap method. *P* value was test using Rao—scott χ^2 considering the variances within provinces. [a] Education level was categorized as bachelor's degree and below and graduate degree and above.

© 2024 Liu H et al. *JAMA Network Open.*

eTable 5. Does-response Association between the Frequency of AI Use and Burnout in Radiology Practice by Subgroups

	Number	Regularly vs. no		Consistently vs. no	
		**OR (95%CI)**	***P* value[a]**	**OR (95%CI)**	***P* value**
Sex					
Male	1797/4350	1.10 (0.97-1.24)	.15	1.68 (1.24-2.28)[b]	.002
Female	878/2376	1.37 (1.15-1.63)[b]	.001	1.35 (0.96-1.91)	.09
Age group					
<40 years	1260/3007	1.23 (1.05-1.43)[b]	.01	1.53 (1.10-2.12)[b]	.01
≥40 years	2304/3719	1.16 (1.01-1.32)[b]	.03	1.48 (1.09-2.01)[b]	.02
Education level [a]					
Low	2135/5502	1.19 (1.07-1.33)[b]	.002	1.57 (1.22-2.02)[b]	<.001
High	540/1224	1.26 (0.97-1.64)	.18	1.10 (0.68-1.77)	.69
Hospital type					
Secondary	990/2738	1.14 (0.96-1.34)	.14	1.42 (0.89-2.28)	.14
Tertiary	1685/3988	1.23 (1.08-1.40)[b]	.003	1.48 (1.15-1.92)[b]	.003
Workload score					
Low score	895/2829	1.09 (0.94-1.27)	.24	1.73 (1.20-2.49)[b]	.007
Medium score	940/2285	1.32 (1.11-1.58)[b]	.003	1.24 (0.87-1.74)	.23
High score	840/1612	1.27 (1.01-1.61)	.05	1.60 (1.00-2.56)[b]	.04
AI acceptance					
Low	629/1504	1.55 (1.24-1.92)[b]	<.001	2.50 (1.33-4.71)[b]	.005
High	2046/5222	1.10 (0.98-1.24)	.09	1.30 (1.02-1.65)	.06

ORs were adjusted for individual and professional characteristics, psychological factors, workload score, and AI acceptance, with the exception of stratified variables, which were mutually adjusted. All analyses were calculated using inverse propensity weighting.

[a] Education level was categorized as bachelor's degree and below and graduate degree and above. [b] *P* < .05 with multiple testing correction by Hochberg method.

AI=artificial intelligence. OR=odds ratio. CI=confidence interval.

© 2024 Liu H et al. *JAMA Network Open.*

eTable 6. Does-response Association between the Frequency of AI Use and Emotional Exhaustion in Radiology Practice by Subgroups

	Number	Regularly vs. no		Consistently vs. no	
		**OR (95%CI)**	**P**	**OR (95%CI)**	**P value**
Sex					
Male	1511/4350	1.08 (0.95-1.23)	.21	1.63 (1.21-2.18)[b]	.002
Female	800/2376	1.38 (1.15-1.64)[b]	<.001	1.37 (0.97-1.93)	.08
Age group					
<40 years	1121/3007	1.24 (1.06-1.45)[b]	.007	1.54 (1.11-2.14)[b]	.002
≥40 years	1190/3719	1.15 (1.00-1.31)[b]	.04	1.43 (1.05-1.94)[b]	.04
Education level [a]					
Low	1822/5502	1.18 (1.06-1.32)[b]	.003	1.55 (1.21-1.98)[b]	.001
High	489/1224	1.33 (1.02-1.73)	.71	1.23 (0.75-2.10)	.41
Hospital type					
Secondary	829/2738	1.06 (0.89-1.25)	.51	1.36 (0.85-2.20)	.41
Tertiary	1482/3988	1.29 (1.13-1.47)[b]	<.001	1.52 (1.18-1.96)[b]	.001
Workload score					
Low score	724/2829	1.07 (0.91-1.25)	.40	1.53 (1.06-2.21)[b]	.04
Medium score	824/2285	1.30 (1.10-1.54)[b]	.005	1.23 (0.87-1.74)	.24
High score	763/1612	1.35 (1.07-1.69)[b]	.02	1.74 (1.10-2.75)[b]	.02
AI acceptance					
Low	522/1504	1.50 (1.21-1.86)[b]	<.001	2.68 (1.43-5.02)[b]	.002
High	1759/5222	1.12 (0.99-1.25)	.06	1.26 (0.99-1.60)	.06

ORs were adjusted for individual and professional characteristics, psychological factors, workload score, and AI acceptance, with the exception of stratified variables, which were mutually adjusted. All analyses were calculated using inverse propensity weighting.

[a] Education level was categorized as bachelor's degree and below and graduate degree and above. [b] $P < .05$ with multiple testing correction by Hochberg method.

Abbreviation: AI=artificial intelligence. OR=odds ratio. CI=confidence interval.

© 2024 Liu H et al. *JAMA Network Open.*

eTable 7. Does-response Association between the Frequency of AI Use and Depersonalization in Radiology Practice by Subgroups.

	Number	Regularly vs. no		Consistently vs. no	
		**OR (95%CI)**	***P* value**	**OR (95%CI)**	***P* value**
Sex					
Male	905/4350	1.06 (0.93-1.20)	.37	1.84 (1.40-2.41)[b]	<.001
Female	309/2376	0.96 (0.79-1.18)	.72	1.24 (0.83-1.86)	.58
Age group					
<40 years	541/3007	1.03 (0.87-1.21)	.75	1.48 (1.07-2.05)[b]	.03
≥40 years	673/3719	1.08 (0.94-1.25)	.27	1.72 (1.27-2.34)[b]	.001
Education level [a]					
Low	1013/5502	1.09 (0.97-1.23)	.13	1.68 (1.32-2.15)[b]	.002
High	201/1224	0.90 (0.69-1.20)	.51	1.19 (0.73-1.95)	.51
Hospital type					
Secondary	496/2738	1.06 (0.89-1.27)	.53	1.37 (0.85-2.20)	.39
Tertiary	718/3988	1.06 (0.92-1.22)	.40	1.60 (1.24-2.05)[b]	<.001
Workload score					
Low score	469/2829	1.06 (0.90-1.25）	.50	1.89 (1.29-2.77)[b]	<.001
Medium score	409/2285	1.14 (0.95-1.37)	.16	1.35 (0.93-1.95)	.16
High score	336/1612	0.94 (0.76-1.17)	.60	1.71 (1.14-2.58)[b]	.02
AI acceptance					
Low	263/1504	1.43 (1.13-1.79)[b]	.005	1.45 (0.81-2.58)	.22
High	951/5222	0.97 (0.86-1.10)	.63	1.54 (1.22-1.96)[b]	<.001

Models were adjusted for individual and professional characteristics, psychological factors, workload score, and AI acceptance, with the exception of stratified variables, which were mutually adjusted. All analyses were calculated using inverse propensity weighting.

[a] Education level was categorized as bachelor's degree and below and graduate degree and above. [b] $P < .05$ with multiple testing correction by Hochberg method.

Abbreviation: AI=artificial intelligence. OR=odds ratio. CI=confidence interval.

© 2024 Liu H et al. *JAMA Network Open.*

eTable 8. Joint Association of AI use, Workload Score, and AI Acceptance with Emotional Exhaustion

Characteristics	Case/n	PR (95%CI)	P value	RERI (95%CI)	RPR (95%CI)
**Workload score**					
Low workload score					
Non-AI group	509/1974	1 (ref)	..	..	
AI group	215 /855	1.07 (0.97-1.19)	.18	..	
Medium workload score					
Non-AI group	386/1116	1.27 (1.15-1.41)	<.001	..	..
AI group	438/1169	1.48 (1.34-1.63)	<.001	0.13 (-0.04 to 0.29)	1.08 (0.94-1.24)
High workload score					
Non-AI group	276/619	1.60 (1.44-1.77)	<.001	..	..
AI group	487/993	1.81 (1.63-2.00)	<.001	0.14 (-0.06 to 0.33)	1.05 (0.91-1.22)
**AI acceptance**					
Low acceptance					
Non-AI group	347/1064	1 (ref)			
AI group	/440/1504	1.28 (1.14-1.44)	<.001	..	..
High acceptance					
Non-AI group	824/2645	1.09 (0.98-1.20)	.10	..	..
AI group	935/2577	1.17 (1.06-1.30)	.004	-0.20 (-0.36 to -0.03)	0.84 (0.73-0.96)

Prevalence ratios were calculated for the joint exposure of AI use and workload score groups by binomial-log regression, adjusting for individual and professional characteristics and psychological factors. All analyses were calculated using inverse propensity weighting. RERI was estimated using the following formula:PR11-PR10-PR01+1. RPR: PR11/(PR01 × PR10). *P* value was corrected by Hochberg method.

Abbreviation: PR=prevalence ratio. AI=artificial intelligence. RERI=relative excess risk due to interaction. RPR=ratio of prevalence ratio. CI=confidence interval.

© 2024 Liu H et al. *JAMA Network Open.*

eTable 9. Joint Association of AI Use, Workload, and AI Acceptance with Depersonalization

Characteristics	n/N (%)	PR (95%CI)	*P* value	RERI (95%CI)	RPR (95%CI)
**Workload score**					
Low workload score					
Non-AI group	629/1974	1 (ref)	..	..	
AI group	266/855	1.11 (0.98-1.27)	.20	..	
Medium workload score					
Non-AI group	444/1116	1.07 (0.93-1.23)	.33	..	..
AI group	496/1169	1.21 (1.06-1.39)	.01	0.03 (-0.17 to 0.23)	1.02 (0.85-1.23)
High workload score					
Non-AI group	309/619	1.27 (1.10-1.47)	.004	..	..
AI group	531/993	1.26 (1.09-1.46)	.006	-0.13 (-0.36 to 0.11)	0.89 (0.73-1.08)
**AI acceptance**					
Low acceptance					
Non-AI group	404/1064	1 (ref)			
AI group	225/440	1.36 (1.14-1.61)	<.001	..	..
High acceptance					
Non-AI group	978/2645	1.24 (1.08-1.44)	.003	..	..
AI group	1068/2577	1.27 (1.10-1.47)	.002	-0.33 (-0.58 to -0.08)	0.75 (0.62-0.91)

Prevalence ratios were calculated for the joint exposure of AI use and workload score groups by binomial-log regression, adjusting for individual and professional characteristics and psychological factors. All analyses were calculated using inverse propensity weighting. RERI was estimated using the following formula:PR11-PR10-PR01+1. RPR: PR11/(PR01 × PR10). P value was corrected by Hochberg method.

Abbreviation: PR=prevalence ratio. AI=artificial intelligence. RERI=relative excess risk due to interaction. RPR=ratio of prevalence ratio. CI=confidence interval.

© 2024 Liu H et al. *JAMA Network Open.*

eTable 10. Association of AI Use with Burnout Adjusting for Each Workload Factor in Regression Models

	**Model 1: Multivariable analysis**	**Model 2: Inverse probability weighting**	**Model 3: Propensity score matching**
AI use in practice, vs. Never or irregularly	1.18 (1.05-1.32)[b]	1.13 (1.02-1.25)[b]	1.18 (1.03-1.35)[b]
Age (per one year increase)	0.99 (0.98-1.01)	1.00 (0.99-1.01)	0.99 (0.99-1.01)
Sex, male vs. female	1.46 (1.29-1.64)[b]	1.52 (1.37-1.69)[d]	1.48 (1.27-1.73)[b]
Geographic regions, vs. West			
East	1.23 (1.08-1.40)	1.18 (1.03-1.35)[b]	1.21 (1.02-1.43)[b]
Central	1.02 (0.86-1.19)	1.04 (0.88-1.24)	1.06 (0.86-1.31)
Northeast	1.10 (0.91-1.31)	0.98 (0.81-1.19)	1.09 (0.86-1.39)
Child, yes vs. no	0.87 (0.73-1.02)	0.86 (0.73-0.99)	0.86 (0.69-1.06)
Income, <5000 vs >5000	1.08 (0.94-1.25)	1.09 (0.96-1.24)	1.19 (0.99-1.44)
Education, high vs. low [a]	1.05 (0.90-1.23)	1.12 (0.97-1.29)	1.12 (0.91-1.36)
Hours per week, vs. <40 hours			
40-49 hours	0.88 (0.76-1.02)	0.88 (0.78-1.00)	0.97 (0.80-1.17)
50-59 hours	1.44 (1.21-1.70)[b]	1.47 (1.26-1.72)[b]	1.65 (1.33-2.06)[b]
60 hours or more	2.02 (1.66-2.46)[b]	2.17 (1.78-2.65)[b]	2.33 (1.80-3.02)[b]
Number of images reading per day, vs. <70			
70-179	1.20 (1.05-1.38)[b]	1.16 (1.03-1.32)[b]	1.17 (0.96-1.42)
180 or more	1.77 (1.50-2.08)[b]	1.72 (1.48-2.00)[b]	1.73 (1.39-2.16)[b]
Hospital grade, tertiary vs. seconday or low	1.15 (1.01-1.30)[b]	1.25 (1.12-1.39)[b]	1.16 (0.99-1.36)
Working equipment, MRI or CT vs. others	1.28 (1.14-1.44)[b]	1.26 (1.14-1.41)[b]	1.29 (1.11-1.50)[b]
Autonomy, disagree or neutral vs. agree	1.69 (1.47-1.94)[b]	1.60 (1.38-1.84)[b]	1.73 (1.44-2.09)[b]
Less control, disagree or neutral vs. agree	1.37 (1.17-1.61)[b]	1.26 (1.09-1.46)[b]	1.38 (1.12-1.70)[b]
Meaningful work, disagree or neutral vs. agree	1.49 (1.27-1.75)[b]	1.51 (1.30-1.75)[b]	1.37 (1.12-1.68)[b]
Reward from work, disagree or neutral vs. agree	0.91 (0.80-1.04)[b]	0.90 (0.80-1.02)	0.90 (0.76-1.07)
Effective support staff, disagree or neutral vs. agree	1.28 (1.12-1.44)[b]	1.35 (1.18-1.54)[b]	1.19 (0.99-1.42)
Effective support facility, disagree or neutral vs. agree	1.41 (1.21-1.65)[b]	1.34 (1.17-1.55)[b]	1.58 (1.29-1.94)[b]

[a] Education level was categorized as bachelor’s degree and below and graduate degree and above. [b] $P < .05$ with multiple testing correction by Hochberg method.

Abbreviation: AI=artificial intelligence. MRI=magnetic resonance imaging. CT=computerized tomography.

© 2024 Liu H et al. *JAMA Network Open.*

eTable 11. Association of AI Use with Burnout among Radiologists by Geographic Locations

	Eastern China		Central China		Western China		Northeast China	
	OR (95%CI)	*P* value [a]	OR (95%CI)	*P* value [a]	OR (95%CI)	*P* value [a]	OR (95%CI)	*P* value [a]
**Burnout**								
AI group vs. Non-AI group	1.14 (0.98-1.34)	.09	1.41 (1.12-1.78)	.003	1.08 (0.91-1.29)	.37	1.33 (1.01-1.75)	.04
Regular vs. never or infrequent	1.11 (0.95-1.30)	.20	1.39 (1.10-1.76)	.01	1.05 (0.88-1.26)	.57	1.33 (0.99-1.77)	.11
Consistent vs. never or infrequent	1.44 (1.01-2.05)	.08	1.75 (0.95-3.25)	.07	1.38 (0.91-2.09)	.27	1.34 (0.82-2.20)	.24
**Emotional exhaustion**								
AI group vs. Non-AI group	1.15 (0.98-1.34)	.08	1.50 (1.19-1.90)	<.001	1.08 (0.90-1.29)	.40	1.36 (1.01-1.76)	.04
Regular vs. never or infrequent	1.08 (0.92-1.27)	.34	1.49 (1.18-1.89)	.002	1.07 (0.89-1.29)	.45	1.33 (0.99-1.78)	.11
Consistent vs. never or infrequent	1.69 (1.17-2.44)	.01	1.65 (0.90-3.01)	.10	1.10 (0.73-1.67)	.64	1.89 (0.83-2.25)	.21
**Depersonalization**								
AI group vs. Non-AI group	1.09 (0.92-1.27)	.34	1.17 (0.92-1.49)	.20	1.04 (0.86-1.26)	.67	1.13 (0.85-1.51)	.40
Regular vs. never or infrequent	1.03 (0.87-1.22)	.71	1.11 (0.87-1.42)	.41	1.00 (0.81-1.21)	.95	1.01 (074-1.39)	.93
Consistent vs. never or infrequent	1.39 (0.99-1.94)	.12	2.11 (1.19-3.75)	.02	1.48 (0.97-2.27)	.14	2.82 (1.07-3.11)	.06

Models were adjusted for individual and professional characteristics, psychological factors, workload score, and AI acceptance, with the exception of geographic location. All analyses were calculated using inverse propensity weighting. [a] *P* value was corrected by Hochberg method for multiple testing.

Abbreviation: AI=artificial intelligence. OR=odds ratio. CI=confidence interval.

© 2024 Liu H et al. *JAMA Network Open.*
