# Supplementary material for: Artificial Intelligence and Radiologist Burnout
Source: JAMA Netw Open. 2024 Nov 22;7(11):e2448714. doi: 10.1001/jamanetworkopen.2024.48714 (PMC11584928; doi:10.1001/jamanetworkopen.2024.48714)
Supplement: Supplement 2. — Data Sharing Statement [file jamanetwopen-e2448714-s002.pdf]

Data Sharing Statement

Liu. Artificial Intelligence and Radiologist Burnout. *JAMA Netw Open*. Published November 22, 2024. doi:10.1001/jamanetworkopen.2024.48714

Data
Data available: No
